# Experience of NHS diagnostic investigation following a multi-cancer early detection (MCED) screening test: qualitative interviews with NHS-Galleri trial participants who had a cancer signal detected

**DOI:** 10.1016/j.eclinm.2025.103733

**Published:** 2026-01-08

**Authors:** Laura A.V. Marlow, Ninian Schmeising-Barnes, Jo Waller

**Affiliations:** Centre for Cancer Screening, Prevention and Early Diagnosis, Wolfson Institute of Population Health, Queen Mary University of London, London, E1 1HH, UK

**Keywords:** MCED, MCD, Liquid biopsy, Follow-up, Investigation, Diagnostic, Navigation, Patient information, Support, Communication, Health service, Trial

## Abstract

**Background:**

Understanding experiences of diagnostic investigation for any new screening modality is important to inform the development of pathways for future implementation. We explored experience of diagnostic work-up within the NHS in people with a cancer signal detected result from a blood-based multi-cancer early detection (MCED) screening test in the NHS-Galleri trial (NCT05611632).

**Methods:**

A subset of 41 participants with a cancer signal detected result (with/without a cancer diagnosis), were interviewed 6-months after their result. Participants described their experiences of diagnostic investigation within the NHS. Reflexive Thematic Analysis was used.

**Findings:**

The journey through the period of diagnostic investigation was extremely varied since this was dependent on the predicted cancer site(s). Participant narratives demonstrated wide variation in the required tests, number of contacts with healthcare staff and duration of the whole process. Five themes were interpreted from participants' narratives: i) Feeling prepared for procedures; ii) Needing to advocate; iii) Needing to self-navigate: iv) Speed of the diagnostic process and having to wait; v) Reaching ‘the end’ of diagnostic work-up.

**Interpretation:**

If MCED screening is implemented in future, it will be important to carefully consider implementation of appropriate diagnostic investigation for patients who have a cancer signal detected. We recommend minimising the length of the diagnostic testing period, offering patient navigation and formulating clear plans for what happens at the end of the patient journey. While our findings highlight important considerations to support positive experiences for those having follow-up tests after a cancer signal detected result, they also have broader application for improvement of cancer diagnostic pathways more generally.

**Funding:**

This work was funded and sponsored by GRAIL Bio UK, Ltd. as a sub-study within the NHS-Galleri trial. GRAIL funded the costs of the data collection as well as staff salaries through a contract with King's College London/10.13039/100009148Queen Mary University of London.

**Research in context**

**Evidence before this study**

Blood-based multi-cancer early detection (MCED) tests are currently being explored as a new possibility for cancer screening. These tests identify markers in the blood that indicate there may be a cancer in the body. The predicted site of the cancer is used to direct follow-up tests. Unlike single-site cancer screening, the multi-cancer aspect of MCED screening means follow-up can be varied. Careful consideration of what follow–up pathways look like and how these are explained to and experienced by patients is vital prior to any implementation.

**Added value of this study**

This is the first study to explore the experiences of people directed into an NHS diagnostic pathway following a cancer signal detected result from an MCED screening test. We give examples of diagnostic pathways as described by participants, highlight prominent themes from participant narratives and make recommendations.

**Implications of all the available evidence**

There are several ways to support positive experiences of diagnostic investigations following a cancer signal detected result from a MCED screening test. These include minimising the time patients are ‘having investigations’, offering patient navigation throughout, ensuring swift communication of ‘no cancer found’ results and providing an opportunity for continued support at the end of the diagnostic journey. While these findings highlight ways to support positive experiences for those having follow-up tests after an MCED blood test, they also reflect more generally how cancer diagnostic pathways can be improved.

## Introduction

Every year thousands of people receive cancer screening results that require further investigation. In 2023–2024 in England, 68,700 women were referred for diagnostic assessment following breast screening^1^; 251,251 women were referred for colposcopy following cervical screening^2^ and 85,612 men and women went on to have a diagnostic test following bowel screening.^3^ There is extensive evidence suggesting that being recalled or referred following an ‘abnormal’ screening result can cause negative psychological outcomes for some people.^4^, ^5^, ^6^

Positive patient experience of tests and procedures is important for reducing the risk of negative psychological outcomes.^7^, ^8^, ^9^, ^10^ The follow-up testing part of the screening process can be broken down into three stages: waiting for follow-up tests, having these tests, and receiving the results. In some cases, treatment or intervention can be within the same ‘see and treat’ appointment (i.e. removal of polyps at colonoscopy); in other cases, additional appointments are required. Qualitative studies suggest that concerns while waiting for follow-up tests revolve around uncertainty and worry about the test procedures, what has caused the ‘abnormality’, fear of cancer, and whether things could get worse while waiting.^11^, ^12^, ^13^, ^14^ Concerns about procedures themselves are often test-specific and include worry about discomfort (e.g. for colposcopy) and the need for sedation/bowel preparation (for colonoscopy^10^). The experience of the follow-up tests themselves can vary given the different procedures but common factors influencing experience include how invasive the tests are,^6^ how prepared people feel,^7^^,^^8^ rapport with health professionals, and needing to self-advocate.^8^ Having to wait for appointments or wait while at the hospital^15^^,^^16^ can also impact experience.

Blood-based multi-cancer early detection (MCED) tests are being explored as a new possibility for cancer screening. These tests identify a ‘signal’ in the blood that indicates there may be a cancer in the body.^17^^,^^18^ MCED testing for asymptomatic people, offered as cancer screening, would involve having a blood sample taken, followed by diagnostic investigation directed towards the predicted origin of the cancer signal. There are still unanswered questions about the use of MCED tests for population-based screening, including their clinical utility, the procedures for confirming a ‘positive’ (cancer signal detected) result, and how much diagnostic investigation/longer term follow-up is tolerable to patients and society more generally.^19^^,^^20^

For single cancer screening tests, follow–up pathways are well-defined (e.g. colonoscopy for bowel cancer screening; colposcopy for cervical) and communicated alongside the screening invitation. Unlike single-site cancer screening, the multi-cancer aspect of MCED screening means it will not be possible to give specific details of what follow-up would involve if someone has a cancer signal detected. Consequently, the follow-up process may be closer to the experience of those referred for routine or urgent investigation after presenting symptomatically in primary care, than standard screening pathways. In a scoping review which included 31 studies exploring patient experiences in the period between presenting with symptoms and first visit to secondary care,^21^ common themes included adequacy of information about the suspected condition and diagnostic tests, feelings of anxiety during the period while waiting, a sense of urgency, and wanting a clear plan for investigation. Other studies exploring patient experience of diagnostic testing suggest complex and varied pathways can feel daunting,^22^ procedures can be more painful than expected^23^ and recall of communications can be a challenge.^24^ Some of these themes will likely be relevant to the experience of undergoing diagnostic testing when a cancer signal is detected following an MCED test.

Understanding experiences of diagnostic investigation for any new screening modality is important to inform the development and communication of pathways for future implementation. This study aimed to explore the experiences of men and women who were referred into the NHS for diagnostic investigation following a cancer signal detected result from a blood-based MCED test as part of the NHS-Galleri trial (NCT05611632).

## Methods

### Design and trial context

The data presented here were collected within the qualitative element of the Psychological Impact of the Galleri Test (sIG(n)al) project,^25^ a substudy embedded within the NHS-Galleri trial.^17^ The NHS-Galleri trial is a randomised controlled trial of an MCED test which looks at methylation patterns on cell-free DNA (the Galleri® test; GRAIL, Inc., Menlo Park, CA, USA). The test detects a cancer signal and predicts the tissue type or organ associated with the signal (cancer signal origin; CSO). In some cases, two CSOs were predicted. Participants within the intervention arm of the trial who had a cancer signal detected were referred into an appropriate NHS urgent suspected cancer referral pathway (depending on CSO) for diagnostic investigation, the same as the symptomatic urgent referral pathway from primary care. The process for referral is described elsewhere.^26^

### Participants

Participants in the NHS-Galleri trial were asymptomatic adults aged 50–77 years, with no history of invasive cancer in the 3-years prior to enrolment.^17^ All participants with a cancer signal detected result in the first year of the trial were sent a questionnaire 6-months after their result (return rate = 72%). Interest in being interviewed was indicated within the questionnaire (68% of those who completed the questionnaire indicated interest in an interview). Interviewees were purposefully selected to represent a range of characteristics, based on their questionnaire data. A sampling frame was used to ensure equal representation of those who self-reported a cancer diagnosis following diagnostic work-up and those who self-reported that their diagnostic work-up did not find cancer (recruitment target n = 20 in each group). Within each of these groups, roughly equal numbers of participants were selected to be male/female; 50–65/66–77 years old and to live in areas of high/low deprivation based on Index of Multiple Deprivation (IMD)^27^ with IMD deciles 1–5 considered low and deciles 6–10 considered high. We made a maximum of three attempts to contact selected participants by telephone, with a voice message left on the final attempt.

### Procedure

Written consent was obtained before the interview. Participants were given the option of a telephone, face-to-face or video call interview. Noone else was present at the interview. Interviews were carried out by researchers with training and experience in qualitative research methods (LM and NSB). Participants were not known to the researchers prior to study commencement. All interviews were audio recorded. Following the interview, participants were given a £40 voucher to thank them for taking part. Interviews were semi-structured and designed to explore experiences and understanding of their MCED test results, referral and diagnostic resolution. This paper focuses specifically on discussions about the experience of having follow-up tests within the NHS. A separate manuscript presents themes related to how participants made sense of their results.^28^

### Materials

Interviews were semi-structured and followed a topic guide (available here: https://osf.io/cau6p) developed by the research team with support from a Patient and Public Involvement (PPI) panel. The order of the discussion was driven by the participant. After the first three interviews, the approach was adapted to start by inviting participants to explain their experience in their own words. This helped to encourage a more open discussion.

### Ethics approval and consent to participate

Ethics approval was granted by the Wales Research Ethics Committee as part of the NHS-Galleri trial (Ref 21/WA/0141). Full consent for the interview was collected prior to participation. This study was performed in accordance with the Declaration of Helsinki.

### Methodological approach and reflexivity

As researchers with backgrounds in psychology, we consider ourselves to be critical realists. We consider attitudes, beliefs and experience of cancer screening and cancer diagnoses to be complex concepts, influenced by social and political contexts. We believe that people will perceive and experience having a cancer signal detected in different ways and make meaning of their subjective reality. We believe we can capture, interpret and explain multiple participant realities using a non-positivist qualitative approach.^29^ LM had previous experience of conducting interviews in the cancer screening context but not with people who had experienced an abnormal result or a cancer diagnosis. At the time of data collection LM, NSB and JW were a senior researcher, research assistant and reader in cancer behavioural science respectively. All are female. LM and JW had PhDs in health psychology and NSB had an MSc in health psychology. LM wrote a reflexive statement before the study started reflecting on her pre-existing conceptions and kept notes throughout documenting the experience of being the interviewer and noting ideas about themes.

### Analysis

Recordings were transcribed verbatim by an external agency. All transcripts were checked by LM/NSB and identifying details (names, places, hospitals or dates) were removed. Reflexive Thematic Analysis^30^ was used as an analysis method. NVivo 1.7 was used to manage the data. LM familiarised herself with the transcripts by reading/re-reading each one and constructing participant timelines. Initial codes were created and refined. Mind-mapping was used to organise these codes into initial themes. Themes were refined. The themes were presented and discussed in detail with NSB and JW. The methods and results of this study are reported following the consolidated criteria for reporting qualitative research (COREQ) checklist.^31^

### Role of the funding source

This work was funded and sponsored by GRAIL Bio UK, Ltd. as a sub-study within the NHS-Galleri trial. GRAIL funded the costs of the data collection as well as staff salaries through a contract with King's College London/Queen Mary University of London. The funder played no role in the design, conduct, analysis or interpretation of the findings but did have the opportunity to review a draft of the manuscript prior to submission. The comments did not alter the findings or interpretation in any way and solely included editorial suggestions to wording to support the clarity of reporting in relation to the trial as well as suggestions for where more detail or additional quotes may be helpful.

## Results

### Overview

**Fig. 1:**
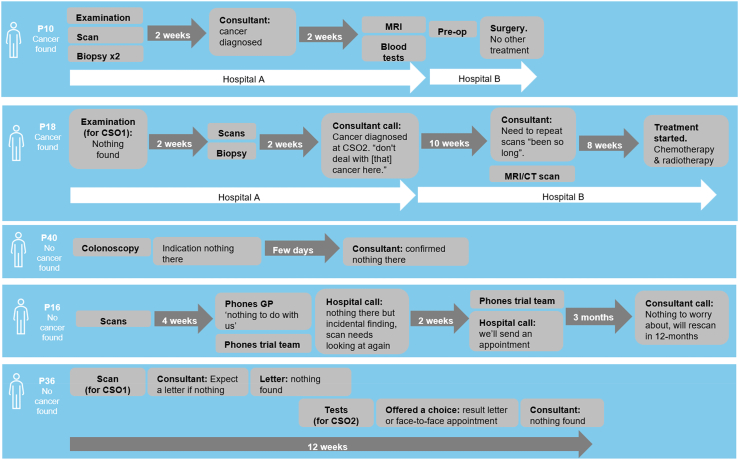
**Example narratives that illustrate variation in the NHS diagnostic pathway for those who went on to have cancer found and those who did not.** CSO is cancer signal origin, the predicted tissue type or organ associated with the cancer signal.

Interviews were completed with 41 participants (see Table 1). Interviews were carried out on the telephone (n = 36) or face-to-face (n = 5) and lasted 22–80 min (mean = 43 min). As expected, the journey through the period of diagnostic testing was extremely varied since this was dependent on the predicted CSO(s). Examples of these journeys, as explained by the participants, are presented in Fig. 1. Participant narratives demonstrated wide variation in the required tests, number of contacts with healthcare staff and the duration of the whole process. There were examples of more and less complex journeys both for those who went on to have cancer found and those who did not. For example, P40 described needing just one visit with one test (colonoscopy) following which, a diagnostic decision was communicated a ‘few days’ later. Conversely, P18 describes needing many different scans and tests across multiple visits and different hospitals.

**Table 1:** Characteristics of interview participants (n = 41).

	Overall (n)
Biological sex	
Male	20
Female	21
**Age**	
≤64	14
65–74	22
≥75	5
**IMD quintile**	
1 (most deprived)	8
2	8
3	6
4	11
5 (least deprived)	8
**Ethnicity**	
White British	37
Mixed ethnic background	1
Any Asian background	2
Any Black background	1
**Marital status**	
Single, never married	3
Married, in a civil partnership or living together	29
Divorced or separated	6
Widowed, not remarried	3
**Highest level of education**	
Finished school at or before the age of fifteen	4
CSEs, O-levels or equivalent	9
A-levels or equivalent	2
Further education but not a degree	12
Bachelor's degree or equivalent	10
Further degree e.g. Masters or PhD	4
**Self-reported cancer diagnosis**	
Yes	21
No	20

Five themes were interpreted from participants' narratives: i) Feeling prepared for procedures; ii) Needing to advocate; iii) Needing to self-navigate iv) Speed of the diagnostic process and having to wait; v) Reaching ‘the end’ of diagnostic work-up. Supporting quotes are reported in the text and in Table 2. Examples for each theme were seen in participants who went on to have cancer found and those who did not, but those without cancer described their diagnostic experience in more detail and consequently there are more supporting quotes from this group.

**Table 2:** Additional Illustrative quotes for each theme.

**Feeling prepared for procedures**
*“Well, I've actually had an MRI scan previous to that”* (P13, no cancer found) *“I'd had one before. I had a colonoscopy before. And I'm not even -- I can't even -- years and years ago. So this time, they said we're going further up the intestine, they gave me a sedative. So, I was relaxed and everything. I didn't feel a thing. So it didn't worry me. It was okay”* (P40, no cancer found). *“And so I've had cameras up and down a little bit before that … so I knew what to expect, and I think it was a camera down if I remember correctly, just the one rather than both. And so I pretty much knew what the drill was, you know, so yeah, but I don't look forward to it, but it's fine”* (P7, no cancer found). *“I had no problem. Because I had had them all before at some stage, similar sorts of things before I mean”* (P8, no cancer found) *“I asked them, I said, If I have a local one, will that be painful? They said, No, no, no, it's fine. So I opted for it, but it was like hell from heaven once I had it, it was really, really painful”* (P24, no cancer found).
**Needing to advocate**
*“But I said, is there somewhere else where they've done something like this? He said, How can there be, we are the best, there's nowhere. So I found him to be very, he was rude and pompous”* (P24, no cancer found) *“You know she says, you're not on the list and starts banging on about this, that and the other. And then I mentioned the Galleri, Don't know anything about that Galleri trial …. I said, There are people coming in here that are absolutely terrified. There are people that come in and they think they've got cancer. They may have cancer. And now you're talking to me like I'm a piece of dirt … it was people looking at me as if to say, Well, who are you sort of thing? Yeah, and at that time I'm the bloke who stood in front of you that may have cancer, what you gonna do about it?”* (P34, no cancer found) *“I went to see a specialist, the specialist was a bit sceptical to be fair, about the trial. He said, one of his statements was, You've been the victim of a trial then?”* (P27, cancer found). *“I rang a receptionist at my GP's practice, and asked to book an appointment to have my staples removed. And initially, they said I couldn't book the appointment, because it had to be within a set week, and they haven't opened that week yet. So I said, So shall I ring tomorrow? And she said, Yes. And so the next day, I rang and said, I'd like to book an appointment with the nurse to remove my staples, and I said, Which day? And she said, Do you have the staple remover? And I said, No. And she said, Well, then I'm sorry, but I can't book the appointment”* (P1, cancer found).
**Needing to self-navigate**
***Examples of pursuing tests or results*** *“She told me that I was going to have this scan. And then, I waited a week. I think I waited two weeks not having heard anything, and I rang them to ask if the results had come through and I spoke to a different person and they said, Yes, it's come through, and a polyp has been found”* (P31, no cancer found) *“What's going on here? So I rung my doctors, and I said, Have you got the results through for, the CT scan? Nothing to do with us, we didn't organise it. And basically put the phone down on me, which I was really, really annoyed about”* (P16, no cancer found) ***Examples of feeling equipped to make contact if needed*** *“It did say that if you do not get appointment with your consultant or a GP within two weeks of us writing to you, you can call us and they would prompt it then, you know, that was really good as well. It's not just saying to us and saying, well, you're going to get the appointment if you don't get in. Luckily, I got the appointments very quick”* (P39, cancer found) *“if I got any concerns or queries or questions not to hesitate to call. So to me, that was fine because whenever I did ring [The Galleri line], I got through. I didn't get press one for this and press two and all that business and, we're busy right now and can't take your call and all that. So to me, that was reassuring and I was quite happy about it. I felt, that if I'd wanted support, I could have contacted you and asked what I should do”* (P42, no cancer found). *“… every stage I was given, even when I was referred back to the NHS, the nurse there who contacted me, she gave me her number, and said, if you've got any questions, give me a ring and, you know, I've got numbers and things I could use if I needed to”* (P7, no cancer found) *“And the nurses at the clinic that were dealing with it gave me a number to get in hold of them if I needed them to answer any questions, and that was it”* (P40, no cancer found)
**Speed of the diagnostic process and having to wait**
***Generally feeling the process was fast*** *“The speed with which I have gone through this journey as well has been incredible, every day I was getting a phone call or a letter to do this or do this, or go there or come here.”* (P25, cancer found) “*Things kicked in very rapidly with contact from the hospital as well.”* (P28, no cancer found) *“My biggest concern at that time was how long will it take [HOSPITAL A] to contact me to do the full investigation. I was blown away by how quickly everything happened.”* (P32, cancer found) *“… within an hour of her coming off the phone, I had a phone call from the respiratory clinic asking me to go for a scan in three days time. But it then became a bit of a whirlwind, you know … Next minute you've got that phone call, then a couple of hours later, you've got a CT scan booked and you think it's all happened suddenly. But that's the way it should be once they find a signal, you know, to be dealt with so quickly, which meant the results came quickly as well. So that was very efficient, I have to say”* (P29, no cancer found) *“The appointments had been made, and the emphasis like if they don't get to see you within the fortnight, get in touch with them and they will push the matter. And really I had the first consultation within the fortnight. And I was quite, again, I thought, I feel really lucky because if it had gone through the doctor and was not feeling well, it would have been probably a long drawn out process”* (P17, cancer found) *“Nothing seemed to take a long time and everything was very efficient … it all went very quickly, I never had to chase anything up”* (P10, cancer found) ***Examples of NHS expectations*** *“I'm also very grateful to the NHS for sort of fast-tracking me through the system, you know, because you hear so much, so many delays happening, people with cancer and all that, but I can't praise them enough, they were quick actually in my case”* (P30, cancer found) *“I think I did get the results as early as possible. You know, I understand that the pressures on the NHS and there is, you know, it's not, things don't always go as quickly as you want it to go”* (P27, cancer found) *“Very confident that things were moving into -- at that this time and at that time still, there's thousands of people waiting on the waiting list for all sorts of things, including cancer. And to think that I was treated so quickly. Obviously, you know, it matters. It matters”* (P31, no cancer found) *“I feel really lucky because if it had gone through the doctor and was not feeling well, it would have been probably a long drawn out process”* (P17, cancer found) *“… eight weeks in the current NHS climate, I think is quite exceptional to be honest”* (P3, cancer found) ***Waiting causes worry*** *“If it had been longer than you know, I would question that -- well, I would say, Look, you know, you do need to be quicker on response to initial indicators because obviously, it is a worry”* (P33, cancer not found) *“Definitely yeah, well it's worrying time, isn't it? The longer you wait, it's more time thinking, Well, what's it going to be?”* (P5, cancer found) *“So I would say it was all very efficient, but it was the anxiety of that say, a two week period of, Is this something I need to be concerned about?”* (P33, no cancer found) *“It all went very quickly, I never had to chase anything up. It was all done and, you know -- I mean at one point I got a phone call on the Wednesday to say, Could I [inaudible] next day to have pre-op. So it was -- nothing took too much time … Because you weren't kind of worrying for months -- I mean I was worrying for months and thinking about it but I wasn't -- there was movement. Things were happening. So I wasn't -- I mean you read in the papers and see on TV at the moment with the backlog that people are waiting six months for appointments”* (P10, cancer found) *“Well, the process of dealing with me has started very quickly. It was a week after I was spoken to … And so if you think of a week with weekend in between, it's pretty quick, and I was impressed with that … [made me feel] very confident that things were moving … Obviously, you know, it matters. It matters.”* (P31, no cancer found)
*“And they had to ring the hospital and they were sort of well, Oh sorry, we sort of forgot about it. Oh yeah, we'll get you booked in within the next few weeks. So I lost another week. When you take that on board of what you've just been told, that other week becomes a year, because your brain can't switch it off, if you can understand what I'm saying.”* (P16, no cancer found) ***Examples of waiting for results (from those who did not have cancer found)*** *“After having the CT scan, it says you'll get the results basically within two weeks. After two weeks, still nothing. Three weeks, nothing. Coming into the fourth week now, and I thought, What's going on here?”* (P16, no cancer found) *“The actual first appointments came quite quickly, but then it was quite a long time after that … It was quite a number of weeks until I was actually told that, No everything was okay. And they had to do a repeat blood test because the results of some white cell count were inconclusive. So in all it took quite a long time, I felt as though it was about 12 weeks it had this anxious period of waiting until somebody actually said to you, No at this time, everything seems to be okay. Can't find anything in our investigations”* (P36, no cancer found) *“So really then the journey, obviously then as you go through and you get appointments and you see a specialist and you go for scans, took a lot longer than I thought it would, and it did seem, was about 12 weeks in total then by the time the scans, etcetera, further blood tests showed that at this time everything was okay”* (P36, no cancer found) *“A bit of a hiatus, you know, a waiting time, because there was no clarity as to whether, what was going to come out. So it was a matter of waiting.”* (P24, no cancer found) **Examples of waiting for results and treatment (from those with cancer)** *“But it would have just been nice, and perhaps someone at [HOSPITAL B] had, rather than just leave you in limbo. Like, We have got all your information, we will get around to seeing you, there is a bit of a backlog, but we are aware, and you are in our system. And, you know, we'll see you as and when we can because, you know, we can afford to wait a few months. That would have been nice to have heard that, if I'm honest.”* (P18 cancer found) *“I've not known really where to turn to because things quite slow moving as well. You can, you've got a cancer nurse but you phoned them up and they'll get back to you within two days. And sometimes they didn't get back to me. So it's, like, sort of very slow … The waiting for results subsequent to that was hard. And just because if you've got, if there's potentially a chance that you do have cancer, you want the results as quick as you can get them so that you can crack on with treatment, you know. So the slowness of how things progressed afterwards was a bit frustrating, but apparently that's part of the course at the moment”* (P26, cancer found) *“I think I did get the results as early as possible. You know, I understand that the pressures on the NHS and there is, you know, it's not, things don't always go as quickly as you want it to go. That's the only thing that would have possibly given me a little bit more comfort”* (P27, cancer found)
*Reaching ‘the end’ of diagnostic work-up*
***Incidental findings*** *“they found two growths on my kidneys … as it turns out, there was nothing, and they said they were benign and shouldn't be a problem at all. And that was the end of that. That line ended there. I mean, that's still something that gives me a bit concern about that. But, however, I didn't have any further investigation”* (P19, no cancer found) ***Feeling in limbo*** *“you picked something up, but at the end of the day, nothing was picked up by anybody anywhere else. So I'm sort of left in a bit of a limbo situation”* (P37, no cancer found) *“Other than being told, you know, there was something there, that didn't unsettle me. What did was, there's something there, and then there wasn't anything there and we kind of left, okay, but now what?”* (P41, no cancer found) *“I thought there had been a bit more explanation and a bit of this and a bit of that, but they were just, oh I'll sign you out, see you later, bye, as if you're like discarded”* (P15, no cancer found)

### Feeling prepared for procedures

Diagnostic investigations involved many different procedures, including some that were considered invasive or uncomfortable. Many participants spoke of prior experiences preparing them for the tests they needed: *“I’ve had colonoscopies before … so I knew what was coming”* (P19, no cancer found). Others were worried when tests were new to them: “*I never had a scan before so I didn’t know what to expect”* (P38, no cancer found).

Some participants who felt unprepared for procedures described feeling scared. For example, P31 was a carer so opted not to have the sedation at colonoscopy, expecting to use gas and air, but said *“I wasn't given that when I went in until the pain was very obvious and I had to shout for it … they weren't prepared to give it to me until I shouted … Now I know, I would tell them to prepare the gas and air for me before I have the procedure started”* (P31, no cancer found). Another example was P5 who was told he had an enlarged lymph node and advised to have a biopsy: *“I was quite shocked at thinking, well how are they going to do this biopsy, what they gonna do, are they gonna cut me open or? And they'd explained that it's a needle … I was thinking, this is going to be really painful”* (P5, cancer found).

### Needing to advocate

Across the diagnostic process there were examples of participants needing to speak up for themselves, including where a participant could not use the hospital gowns because of their size and had to push for a reasonable adjustment or needing pain relief.

Participants recalled positive and negative interactions with healthcare staff at different points in their journey. This included reception staff, those carrying out the tests and consultants (sometimes more than one). Several participants described consultants who were unfamiliar with the NHS-Galleri trial meaning they had to explain why they were there. Some described negative attitudes from healthcare staff and how this made them feel: *“the consultant didn't seem to really know about this trial or maybe had some sort of vague recollection about it … I felt he was a little bit dismissive, to be honest, because obviously I was concerned and anxious”* (P36, no cancer found). There were also examples of participants (early on in the trial) needing to explain why they had been referred when they were asymptomatic: *“… when I saw the doctor for the colonoscopy, he said, so you've had diarrhoea. And I said, no I've got no symptoms. And I had to explain that I had come via the trial … I think later down the process, there'd obviously been more people coming through from the Galleri trial. So I was starting to encounter people who had knew more about it”* (P35, cancer found).

### Needing to self-navigate

Participants described examples of having to navigate their way through the diagnostic testing period including pursuing appointments and results of investigations. Self-navigation was not always a problem when it was clear how to do this: *“[they] said, look, if you don't get the appointments within two weeks, let us know and we'll chase it up for you, which is really good and very, very helpful”* (P39, cancer found). Not knowing the next step without a point of contact to pursue the answer from was considered frustrating. The trial context meant participants who were not sure how to contact the hospital could contact the research nurses who could facilitate communication: *“I rung the Galleri trial. They rung [HOSPITAL A]. And the same afternoon, they rung me back, [HOSPITAL A]. Oh, we've got your results. Why didn't [HOSPITAL A] ring me in the first place, and tell me these results where I've sat there for another two weeks, worrying my socks off, of what's going on?”* (P16, no cancer found).

The transfer over to investigate a second CSO was a point in the journey that some participants described trying to navigate. For example, P28 had a colonoscopy with no polyps found, following which she wanted to clarify what would happen with the second CSO (breast). She described feeling frustrated that the colonoscopy nurse was not able to provide this information: *“that information obviously wasn't available, and she didn't know who I could talk to”*. In an attempt to establish the next step, P28 walked across to the breast department which was in the same hospital: *“they didn't know anything about it. … there was a sort of sense of, okay, so what happens next? And it didn't feel that coordinated.”* (P28, no cancer found). For P28, the communication of results following investigation of the two CSOs also led to confusion when she was asked by doctor B (second CSO) if she had heard from doctor A (first CSO) yet: *“Of course part of my brain was going, Oh, dear, they found something. And [doctor B] clearly thinks [doctor A] should have told me. And part of my brain was going, well if they haven't got around to doing it, then presumably there's nothing dire going on there”* (P28, no cancer found). For P29, pursuit of tests for the second CSO was made through the GP: *“… they rushed through the respiratory tests and I didn't hear anything from the breast clinic. So I went to my GP surgery and showed them the letter where it said, I've got some signals for the lung and breast cancer. I've had lung test, but nobody's contacted me about breast. So my GP contacted the Galleri trial, who then got me to see a breast consultant”* (P29, no cancer found).

The need for navigation to find out results was described by some of those with no cancer found: *“Job got done, up until the point of the CT scan. After that, it was just dead silence, I had to keep on ringing up. Well, are there any results … by this time, I'm pretty interested to hear”* (P34, no cancer found). For those with a cancer diagnosis, some also described needing to self-navigate ahead of receiving treatment: *“I just kept phoning up, really. And I don't know whether we -- I think I got lost in the system if I'm honest”* (P18, cancer found)

### Speed of the diagnostic process and having to wait

Needing to wait caused concern and being seen speedily reduced it. Many felt investigations were quick and efficient: *“the actual experience was actually quite smooth and quite quick, you know, there was no real hanging about”* (P38, no cancer found). Participants commented on how they felt about the speed with which they had their first appointment and completed diagnostic investigation. Many interpreted this within the context of expected waiting times within the NHS which were perceived to be long and so some felt they had been ‘fast tracked’. Getting the tests done quickly limited the period of time that people were left to worry about cancer: “*I'd rather go through them sooner rather than later rather than be, wait for weeks and weeks, get it all over and done … because you're not worrying about it too long. It's not prolonging your own anxiety of what is and what isn't. Get in, get the results and then working it out from there.”* (P38, no cancer found).

For participants who did not have cancer found, some recalled indications that there was ‘nothing there’ during the diagnostic tests; others continued to be alert to cancer as a likely threat until officially being told it was not. Waiting for results following tests was considered a worrying time: *“And then it's just waiting for the results, to then know whether it was or it wasn't so that was the only time I felt a little bit anxious was after I had the op while they did the biopsy on it and found out what it was or what it wasn't.”* (P38, no cancer found).

Some participants felt that the period between their last test/scan and having the results communicated to them was too long, and this extended a period of worry and concern that cancer was a possibility. Several participants reported waiting months for a scheduled appointment, only to be told that there was nothing to worry about: *“So in all it took quite a long time, I felt as though it was about 12 weeks I had this anxious period of waiting until somebody actually said to you, No at this time, everything seems to be okay. Can't find anything in our investigations”* (P36, no cancer found). Participants suggested that communicating a ‘no cancer found’ result should happen as soon as possible: *“I would say that if people were found to be clear, not to let them worry, and, for another month while they're waiting to see a specialist -- get in touch with them. You can get in touch with them, to tell them there's a spike, so surely get in touch with people to say, Look, don't worry, everything's fine”* (P13, no cancer found).

Delays between diagnostic tests and resolution were less commonly described among people with cancer. For those with cancer, concern came with delays prior to starting treatment and these were considered worrying and frustrating. For example, P18 described how *“the consultants said to me at [HOSPITAL A], when he referred me, he said you should hear something within two weeks, and after about four weeks, I started to think, have they forgotten me?”* During this waiting time, P18 was worried that the cancer might spread: *“I don't know how long it takes for it to spread, is it that I'm not those people? And I understand there could have been people needing treatment before me, because they were further advanced, and I will probably, because I was low stage, not be as urgent. But it would have just been nice … rather than just leave you in limbo. Like, We have got all your information, we will get around to seeing you, there is a bit of a backlog, but we are aware, and you are in our system. And, you know, we'll see you as and when we can because, you know, we can afford to wait a few months ….”* (P18, cancer found).

### Reaching ‘the end’ of diagnostic work-up

Perceptions of ‘resolution’ sometimes conflicted with what may have been considered a clinical resolution, with examples of confusion over whether the diagnostic experience had finished. For example P30 described how they thought the investigations were over before being contacted ‘a few weeks later’ regarding investigation for the second CSO: *“… they were looking at the pancreas, and that came back normal … one a few weeks later I had the MRI that also said, you know, they didn't find anything, so I really thought that was the end of it … then I was surprised, actually, when I was contacted again by the lady at [Hospital A] to say that she had been in touch with yourselves, I think, and they thought that she should look at more of the gallbladder and liver area. So we had to have another MRI.”* (P30, cancer found).

For some, cancer was not found at the CSO, but incidental findings required further investigation. For example, P16 did not have cancer found, but the consultant decided to investigate incidental findings—a lung nodule picked up on a scan: “*So all of that time, I'm still sitting there thinking, Have I got lung cancer now? Although it wasn't picked up on your trial, it was picked up on the scan. Until I got that phone call from that consultant, and having the conversation with him sort of gave me a bit more reassurance that there wasn't a major problem”* (P16, no cancer found).

Several participants who did not have cancer found described feeling ‘discarded’ at the end of the diagnostic process: “*The finish was a bit abrupt … I suddenly got a phone call saying you’re, we’re putting you back to your GP and that’s it”* (P19, no cancer found). One participant who did not have cancer found but was told they would be re-scanned described how they felt resolution had not been reached: “*They said, I don't want to let you go completely because something showed up, you know? … we'll just have the scan in 18 months time. It's a strange thing, really, between the two, if you see what I mean?*” (P29, no cancer found).

## Discussion

This paper presents themes from interviews with asymptomatic men and women who had undergone diagnostic follow-up within the NHS, after having a cancer signal detected result from an MCED test. Many of these themes echo qualitative work that has explored experience of further investigation following an abnormal screening result (for cervical, colorectal or breast screening) or symptomatic investigation. This includes concern and feeling prepared for procedures,^12^^,^^13^ finding them more painful than expected,^23^ needing to advocate^8^ and anxiety caused by having to wait.^15^^,^^16^^,^^21^

Speedy return of results from diagnostic investigations was associated with satisfaction whereas periods of waiting were considered difficult, consistent with findings from existing screening programmes.^21^^,^^32^^,^^33^ Anxiety and concern during the diagnostic period following symptoms are thought to be minimised when the time lag is shorter^34^ and consequently the NHS Faster Diagnosis Standard (FDS) places a target of finding out whether you have cancer or not within 4-weeks of referral.^35^ In our study, many of the participants described how quickly they were seen and this led to a positive experience. The gap between the end of testing and communication that no cancer had been found was raised by several participants as being too long and led to sustained concern. Processes and targets should be clearly defined in any future MCED programme to ensure waiting times are limited across the entire pathway, including if more than one CSO is being investigated and when ‘no cancer found’ results are to be delivered. If in future, MCED tests report only one CSO to support focused diagnostic evaluation, this may also bring about better patient experience.

Our work also highlights the importance of having a ‘navigator’ available to patients during the diagnostic journey. This has (understandably) not been a prominent theme in single-site cancer screening research, where pathways are more straightforward. Patient navigation is used across the cancer care continuum and can support care coordination, empowerment, advocacy, information and emotional support.^36^ There is also some suggestion that patients with known navigators are protected from the impact of diagnostic delays.^34^ In the 2016 Care Navigation report,^37^ the navigator role is described as ‘a go to person who glues it all together’. The report focuses on care for chronic illnesses like dementia or diabetes—yet the principles are incredibly pertinent to the experience of diagnostic testing, especially where this extends beyond one visit and result, or across cancer specialities in the case of multiple CSOs following an MCED test. For the NHS-Galleri trial, the people who ‘glued it all together’ were the trial team including co-ordinators, a dedicated patient navigator and research nurses. Observational work within cancer non-specific symptom (NSS) pathways suggests that navigator roles already exist in some sites (but not all), covering management of referrals, organising testing, reporting results and creating a sense of continuity for the patient, explaining and enabling them across the pathway.^38^ Utilising existing navigators may be an option for those under investigation following a positive MCED test result, but the demand for these services should be considered prior to any MCED implementation. Interestingly, rather than being steered through the process by a designated navigator, some participants were incredibly active within their navigation through the diagnostic process—chasing appointments and results. Many described keeping notes and diaries to keep track of what was going on. Having the tools to support self-navigation (contact details, plans for testing) provided reassurance and reduced uncertainty and worry. If MCED screening were implemented, interventions to facilitate self-navigation should be considered for those requiring follow-up following investigation. Reporting of data about the use of NHS services among those with a signal detected result will be an important part of the NHS-Galleri trial. This may include time to diagnostic resolution, number of diagnostic tests required, and utilisation of navigation or further support. These data will help determine the impact on services and inform service design if MCED cancer screening is implemented.

Where further tests were complete and a result of ‘no cancer found’ had been communicated, some participants would have liked additional support. It is possible that this desire for extra support stemmed from higher suspicion of cancer and residual concern.^28^ Previous work suggests that there is unmet need when it comes to reassurance following false positive screening results.^11^ Research exploring cancer diagnostic pathways for symptomatic patients highlights the importance of ‘owning the patient’ until a diagnosis is reached^38^ at which point the responsibility for those with no cancer returns to the GP. In existing screening programmes, patients who have undergone follow-up may have a sense of resolution following treatment (e.g. polyps are removed) or receive more frequent recall through the screening programme (e.g. if HPV-positive). Establishing a longer-term plan for people who have experienced ‘false-positive’ results and how this is best communicated will be an important consideration ahead of any MCED screening programme.

The variation in complexity of participant journeys illustrates how different the post-result experience can be. The NHS cancer screening programmes ‘helping you decide’ leaflets give an overview of the further tests that would be needed following an abnormal result (https://phescreening.blog.gov.uk/leaflets/) in line with informed choice recommendations.^39^^,^^40^ Rather than being able to communicate this pathway ahead of participation in MCED screening, information will need to highlight the uncertainty about what might come next and a need for diagnostic testing to be flexible, depending on the CSO(s). Providing a more general indication of some of the possible procedures and testing options may be possible. Future research exploring how best to do this would be beneficial.

This work is not designed to be a representative picture of experiences; instead we share aspects of participants’ narratives to make recommendations for improvement. Qualitative work that asks people about their experiences of healthcare can result in a retrospective bias—with people remembering specific aspects that they found worrying or upsetting.

The processes for clinical referral to the NHS after a cancer signal was detected in the NHS-Galleri trial were adapted and improved throughout the trial.^26^ A separate paper reporting on clinical referral in the trial highlighted that there were more issues (e.g. lacking awareness of the trial among clinicians) reported in the first year (the year within which we collected these qualitative data), than in the subsequent years.^26^

We purposefully sampled to ensure diversity across different sexes, ages and levels of deprivation. However, we only had a few participants from ethnic minority backgrounds and all spoke English. Further work should consider how patient pathways following abnormal screening results are experienced by those from marginalised groups including ethnic minority populations and non-English speakers, but also people with learning and physical disabilities. This is particularly important given evidence of inequity across the cancer care pathway.^41^

The findings suggest ways to improve patient experience during the diagnostic testing period among people who are undergoing investigations following a cancer signal detected MCED screening test result. The period of time patients are ‘having investigations’ should be minimised, patient navigation should be available, and attention is needed for what happens at the end of the journey, especially for those with no cancer found. While these findings highlight considerations that are needed to support positive experiences for those having follow-up tests after an abnormal MCED test, they also have wider implications for how cancer diagnostic pathways can be improved. With a rapidly evolving landscape in relation to MCED screening tests, we recommend that evaluation of patient experience continues.

## Contributors

Laura A.V. Marlow: conceptualisation, methodology, data curation, formal analysis, interpretation, writing.

Ninian Schmeising-Barnes: data curation, project administration, interpretation, writing.

Jo Waller: conceptualisation, funding acquisition, methodology, supervision, interpretation, writing.

The data had been accessed and verified by all authors.

## Data sharing statement

Due to the sensitive nature of the transcripts these will not be made publicly available.

## Declaration of interests

JW reports research income from GRAIL Bio UK, Ltd., which funded 20% of her salary (2021–2024) and the full salaries of LM (2021–2025) and NSB (2021–2023) through a contract with King's College London/Queen Mary University of London.

## References

[1] Accredited official statistics (2025). https://digital.nhs.uk/data-and-information/publications/statistical/breast-screening-programme/england---2023-24.

[2] Accredited official statistics Cervical Screening Programme, England - 2023-2024 2024. https://digital.nhs.uk/data-and-information/publications/statistical/cervical-screening-annual/england-2023-24#.

[3] NHS England (2025). Bowel Cancer Screening Standards Data Report 2023-24. https://www.gov.uk/government/publications/bowel-cancer-screening-annual-report-2023-to-2024/bowel-cancer-screening-standards-data-report-2023-24.

[4] Quaife S.L., Janes S.M., Brain K.E. (2021). The person behind the nodule: a narrative review of the psychological impact of lung cancer screening. Transl Lung Cancer Res.

[5] Rogstad K.E. (2002). The psychological impact of abnormal cytology and colposcopy. BJOG.

[6] Bond M., Pavey T., Welch K. (2013). Systematic review of the psychological consequences of false-positive screening mammograms. Health Technol Assess.

[7] O'Connor M., Waller J., Gallagher P. (2015). Understanding women's differing experiences of distress after colposcopy: a qualitative interview study. Womens Health Issues.

[8] Gopisetty D.D., Rogers-Shepp I., Padron E., Shankar M., Shaw K.A. (2025). Understanding patient experiences during gynaecological procedures: a qualitative exploratory study. BMJ Sex Reprod Health.

[9] O'Connor M., Gallagher P., Waller J. (2016). Adverse psychological outcomes following colposcopy and related procedures: a systematic review. BJOG.

[10] Restall G., Michaud V., Walker J.R. (2020). Patient experiences with colonoscopy: a qualitative study. J Can Assoc Gastroenterol.

[11] Long H., Brooks J.M., Harvie M., Maxwell A., French D.P. (2019). How do women experience a false-positive test result from breast screening? A systematic review and thematic synthesis of qualitative studies. Br J Cancer.

[12] Toft E.L., Kaae S.E., Malmqvist J., Brodersen J. (2019). Psychosocial consequences of receiving false-positive colorectal cancer screening results: a qualitative study. Scand J Prim Health Care.

[13] Kohler R.E., Hemler J., Wagner R.B. (2023). Confusion and anxiety in between abnormal cervical cancer screening results and colposcopy: "the land of the unknown". Patient Educ Counsel.

[14] Kirkegaard P., Edwards A., Larsen M.B., Andersen B. (2018). Waiting for diagnostic colonoscopy: a qualitative exploration of screening participants' experiences in a FIT-based colorectal cancer screening program. Patient Prefer Adherence.

[15] Bosgraaf R.P., de Jager W.C., Servaes P., Prins J.B., Massuger L.F., Bekkers R.L. (2013). Qualitative insights into the psychological stress before and during colposcopy: a focus group study. J Psychosom Obstet Gynaecol.

[16] Christensen H.M., Huniche L. (2020). Patient perspectives and experience on the diagnostic pathway of lung cancer: a qualitative study. SAGE Open Med.

[17] Neal R.D., Johnson P., Clarke C.A. (2022). Cell-free DNA-based multi-cancer early detection test in an asymptomatic screening population (NHS-Galleri): design of a pragmatic, prospective randomised controlled trial. Cancers (Basel).

[18] Clarke C.A., Mitchell B.L., Putcha G. (2024). Lexicon for blood-based early detection and screening: BLOODPAC consensus document. Clin Transl Sci.

[19] Etzioni R., Gulati R., Weiss N.S. (2022). Multicancer early detection: learning from the past to meet the future. J Natl Cancer Inst.

[20] Eisenstein M. (2025). Putting early cancer detection to the test. Nature.

[21] Wheelwright S.J., Russ S., Mold F., Armes J., Harder H. (2024). Symptomatic presentation of cancer in primary care: a scoping review of patients' experiences and needs during the cancer diagnostic pathway. BMJ Open.

[22] Haste A., Lambert M., Sharp L., Thomson R., Sowden S. (2020). Patient experiences of the urgent cancer referral pathway-can the NHS do better? semi-structured interviews with patients with upper gastrointestinal cancer. Health Expect.

[23] Ndukwe N., Borowski D.W., Lee A., Orr A., Dexter-Smith S., Agarwal A.K. (2012). The two-week rule for suspected colorectal cancer: patient experience and psychological impact. Int J Health Care Qual Assur.

[24] Cornford C.S., Harley J., Oswald N. (2004). The '2-week rule' for suspected breast carcinoma: a qualitative study of the views of patients and professionals. Br J Gen Pract.

[25] Marlow L.A.V., Schmeising-Barnes N., Warwick J., Waller J. (2023). Psychological impact of the Galleri test (sIG(n)al): protocol for a longitudinal evaluation of the psychological impact of receiving a cancer signal in the NHS-Galleri trial. BMJ Open.

[26] Lowenhoff I., Dolly S., Smith R.D. (2025). Clinical referral to the NHS following multi-cancer early detection test results from the NHS-Galleri trial. Front Oncol.

[27] Accredited official statistics (2019). The English Indices of Deprivation 2019 (IoD2019). https://www.gov.uk/government/statistics/english-indices-of-deprivation-2019.

[28] Marlow LAV, Schmeising-Barnes N, Waller J (2025). The impact of cancer expectations on psychological responses following a cancer signal detected result in asymptomatic multi-cancer detection (MCED) testing. Br J Cancer.

[29] Braun V., Clarke V. (2023). Toward good practice in thematic analysis: avoiding common problems and be(com)ing a knowing researcher. Int J Transgend Health.

[30] Braun V., Clarke V. (2021).

[31] Tong A., Sainsbury P., Craig J. (2007). Consolidated criteria for reporting qualitative research (COREQ): a 32-item checklist for interviews and focus groups. Int J Qual Health Care.

[32] Miles A., Wardle J., Atkin W. (2003). Receiving a screen-detected diagnosis of cancer: the experience of participants in the UK flexible sigmoidoscopy trial. Psychooncology.

[33] Posner T., Vessey M. (1988). Psychosexual trauma of an abnormal cervical smear. Br J Obstet Gynaecol.

[34] Brocken P., Prins J.B., Dekhuijzen P.N., van der Heijden H.F. (2012). The faster the better?-A systematic review on distress in the diagnostic phase of suspected cancer, and the influence of rapid diagnostic pathways. Psychooncology.

[35] NHS (2021). Faster diagnosis framework and the faster diagnostic standard. https://www.england.nhs.uk/cancer/faster-diagnosis/.

[36] Chan R.J., Milch V.E., Crawford-Williams F. (2023). Patient navigation across the cancer care continuum: an overview of systematic reviews and emerging literature. CA Cancer J Clin.

[37] Health Education England (2016). Care Navigation: a Competency Framework. https://www.hee.nhs.uk/sites/default/files/documents/Care%20Navigation%20Competency%20Framework_Final.pdf.

[38] Black G.B., Nicholson B.D., Moreland J.A., Fulop N.J., Lyratzopoulos G., Baxter R. (2025). Doing “detective work” to find a cancer: how are non-specific symptom pathways for cancer investigation organised, and what are the implications for safety and quality of care? A multisite qualitative approach. BMJ Qual Saf.

[39] Jepson R.G., Hewison J., Thompson A., Weller D. (2007). Patient perspectives on information and choice in cancer screening: a qualitative study in the UK. Soc Sci Med.

[40] Jepson R.G., Hewison J., Thompson A.G., Weller D. (2005). How should we measure informed choice? The case of cancer screening. J Med Ethics.

[41] Estupinan Fdez de Mesa M., Marcu A., Ream E., Whitaker K.L. (2024). Using the candidacy framework to understand individual, interpersonal, and system level factors driving inequities in women with breast cancer: a cross-sectional study. BJC Rep.

